# Porcine Models of the Intestinal Microbiota: The Translational Key to Understanding How Gut Commensals Contribute to Gastrointestinal Disease

**DOI:** 10.3389/fvets.2022.834598

**Published:** 2022-03-25

**Authors:** Elizabeth C. Rose, Anthony T. Blikslager, Amanda L. Ziegler

**Affiliations:** Department of Clinical Sciences, College of Veterinary Medicine, North Carolina State University, Raleigh, NC, United States

**Keywords:** intestinal microbiota, translational models, pig models, gut commensals, gastrointestinal disease

## Abstract

In the United States, gastrointestinal disorders account for in excess of $130 billion in healthcare expenditures and 22 million hospitalizations annually. Many of these disorders, including necrotizing enterocolitis of infants, obesity, diarrhea, and inflammatory bowel disease, are associated with disturbances in the gastrointestinal microbial composition and metabolic activity. To further elucidate the pathogenesis of these disease syndromes as well as uncover novel therapies and preventative measures, gastrointestinal researchers should consider the pig as a powerful, translational model of the gastrointestinal microbiota. This is because pigs and humans share striking similarities in their intestinal microbiota as well as gastrointestinal anatomy and physiology. The introduction of gnotobiotic pigs, particularly human-microbial associated pigs, has already amplified our understanding of many gastrointestinal diseases that have detrimental effects on human health worldwide. Continued utilization of these models will undoubtedly inform translational advancements in future gastrointestinal research and potential therapeutics.

## The Rise and Plateau of Gut Microbiota Research

Over the first few days of life, the neonatal gut is rapidly populated by a diverse population of microorganisms that exponentially expands to a number exceeding that of the total host cells (1). Interestingly, a recently-proposed paradigm shift suggests that the gut microbiota begins to develop *in utero* rather than during birth (2–5). Such findings are pivotal to our understanding of the gut microbiota given that microbes support vital physiologic processes including production of volatile fatty acids and vitamin K as well as transportation of electrolytes and water across the mucosal surface. The presence of gut commensals is particularly crucial in developing neonates, the developing immune system of which is stimulated by indigenous microflora. Furthermore, the absence of certain gut commensals has been associated with increased risk for lifelong autoimmune diseases (6, 7).

Paralleling increased interest in the gut bacterial microbiota, herein referred to as the gut microbiota, there have been robust advancements in laboratory technologies able to characterize such populations. Increased accessibility to 16S rRNA and next-generation DNA sequencing has permitted detailed characterization of the gastrointestinal microbiota in numerous species. Furthermore, “-omics” laboratory techniques have informed investigations into the systemic impact of gut microbial communities. These communities are now heavily linked with the development of several intestinal and multisystemic diseases in humans including obesity and inflammatory bowel disease (IBD).

Despite instrumental advances in microbial identification, establishing association between variations in microbial diversity and disease phenotypes is continuously challenged by sampling limitations within humans. Subsequently, gastrointestinal research has experienced drastic drops in the development of new pharmaceuticals and diagnostics despite large monetary investments by public and private institutions. This decelerated translation of basic research to clinical practice is colloquially referred to as the “pipeline problem” (8). This problem is attributable, at least in part, to the historic use of inappropriate animal models, frequently rodents, that do not adequately emulate the human patient. Consequently, gastrointestinal research has steadily turned toward pig models given their anatomic and physiologic similarities with humans (9). This review examines these similarities and summarizes recently-characterized parallels between the pig and human gut microbiota, thereby advocating for the pig as an unequaled model of the human gut microbiota and a powerful tool to break through current plateaus in exploring disease association with the gut microbiota.

## Anatomic and Physiological Similarities Between the Human and Pig Outweigh Differences

**Figure 1:**
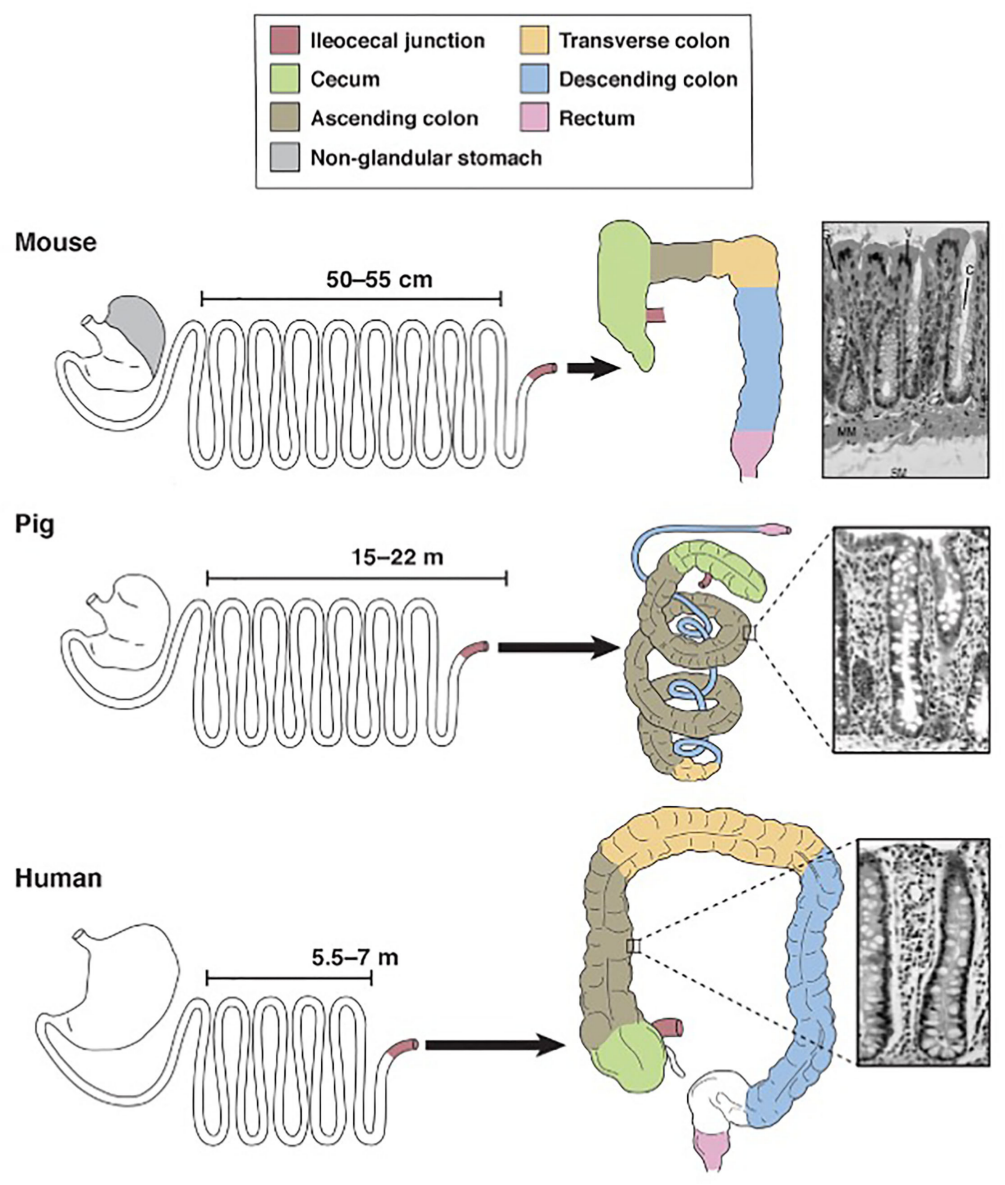
Schematic diagram for comparison of mouse, pig and human gastrointestinal tract anatomy. Derived from Ziegler et al. (13).

Nutritionists around the world continue to preach that we are what we eat. In the realm of gastroenterology, this proverb can be interpreted literally given that gastrointestinal morphology is directly associated with meal frequency, type of food from which nutrients are extracted and composition of the gut microbiota (10, 11). Unlike rats and other domestic animals, the pig and human are both true omnivores (12). Therefore, it comes as no surprise that their gastrointestinal tracts share many macroscopic and microscopic features (Figure 1).

Pigs, rodents and humans all have a “simple” stomach comprised of one compartment. While the entirety of the human stomach and majority of the pig stomach are glandular, the rodent stomach is divided into a glandular portion and non-glandular portion. This non-glandular portion, which is used for food storage and digestion, defines nearly 50% of the gastric mucosal surface area in rodents, therefore complicating the use of rodent models for human gastric studies. In all three species, the glandular section is composed of cardiac, gastric and pyloric mucosa. The pig stomach contains significantly more cardiac mucosa than the human stomach. This cardiac mucosa creates a pseudo-diverticulum for food storage as well as digestion and is a proposed site of microbial metabolism. Furthermore, cardiac epithelial cells are mainly mucus-secreting while parietal and chief cells of the gastric and pyloric mucosa secrete hydrochloric acid and pepsinogen, respectively. Taking these physiochemical differences into account, the relatively large size of the cardiac mucosa in pigs may support a unique, physiologic niche for microbes that is not mirrored in the human. Therefore, caution is urged when making comparisons between human and pig gastric microbiota. Researchers should be aware of this anatomic variation and consider sampling protocols that emphasize collection from the shared gastric and pyloric mucosa rather than the cardiac mucosa.

Aboard to the stomach, porcine and human intestines are strikingly similar. The ratio of total intestinal length per kilogram bodyweight is ~ 0.1 in both pigs and humans, meaning both species share a similar relative length of their intestinal tract (14, 15). The small intestines of both species are macroscopically similar, characterized by a linear, continuous tube anchored to the peritoneum by intricately-vascularized mesentery. This linear morphology is retained in the human large intestines but disrupted in the pig by the formation of a spiral colon that coils into itself. The pig colon is additionally slightly larger than the human colon; the pig colon accounts for approximately 46% of total intestinal tract weight while the human colon accounts for approximately 36% of total intestinal tract weight (14). Despite this discrepancy in shape and relative size, however, the large intestines in both species are functionally similar and house the body's largest population of microbes. Notably, the large intestinal microbiota in both species is responsible for synthesizing volatile fatty acids, which are absorbed through the intestinal mucosa (16). Sacculations and tenia within the human and pig colon additionally provide similar physiologic and anatomic niches for gut commensals and thereby may encourage similar microbial populations.

The colon is also the primary site for ingesta fermentation in both pigs and humans. Rodents, however, are cecal fermenters. Given that intestinal fermentation is primarily regulated by luminal microbes, its anatomic localization directly affects the constituents of the gut microbiota. Therefore, given that rodents ferment within their cecum, the constituents of their large intestinal microbiota will significantly vary from that of colon fermenters, namely pigs and humans. The pig and human ceca do diverge with respect to their size; the pig cecum is relatively large and grossly demarcated from the remainder of the large intestines compared to the human cecum. Therefore, similar to the gastric cardia, the study of the pig cecum as a model for humans must be regarded with caution.

Mucosal Peyer's patches are another anatomic feature that distinguishes the pig and human gastrointestinal tract, specifically the small intestines, from one another. Pig and human Peyer's patches intestines diverge with respect to the cellular composition, development, distribution and number (12, 17, 18). In fact, organogenesis research suggests that pigs develop two distinct types of Peyer's patches, jejunal and ileal, while humans develop only one (19). Given that one of the Peyer's patches many functions is to discriminate between pathogenic and commensal bacteria, we can speculate that this interspecies variability may provoke distinct host perception of and interaction with gut commensals. Conversely, several researchers argue that these differences between pig and human Peyer's patches are of limited significance because pigs and humans demonstrate similar immunologic responses to various gastrointestinal insults (12, 20, 21).

The anatomic similarities between the pig and human gastrointestinal tracts translate to comparable intestinal motility, referring to the contraction and relaxation of the muscularis layers, as well as similar ingesta transit time, referring to the time ingesta takes to travel from the esophagus to the rectum (22). Significant alterations in the gut microbiota are regularly detected in individuals with altered intestinal mobility, such as surgery patients with postoperative ileus. Therefore, similar ingesta transit times between pigs and humans may facilitate similar populations of gut microbiota by discouraging colonization by pathogenic or non-commensal organisms.

## Comparing the Neonatal and Adult Intestinal Microbiota of Pigs and Humans Reveals Important Similarities and Differences

Many studies have made impactful comparisons between the intestinal microbiota of pigs and humans (23–25). Although the intestinal microbiota in healthy adults and mature pigs is relatively stable, it fluctuates widely over the first year of life for both species (26–28). One study has demonstrated that piglets from the same litter as well as newborn human twins can differ with respect to their intestinal microbiota (29). Furthermore, adult humans and pigs demonstrate similar alterations to their gut microbiota in response to environmental stressors and antibiotics (30–35). This suggests that the pig is a powerful model of pathologic disturbances in the intestinal microbiota, such as those elicited by antibiotic-induced dysbiosis and IBD.

Notably, most studies on the intestinal microbiota are based primarily on fecal samples due to limitations in sample acquisition. Sampling the human gastrointestinal microbiota is particularly challenging given that elective surgeries to obtain such samples are costly, time-consuming, and simply unappealing to most individuals. Elective surgeries in laboratory animals are certainly more feasible but still elicit a systemic stress response, which may compromise the microbiota and consequently the study's integrity. Therefore, very few studies have provided direct, interspecies comparisons of microbial populations across specific sites of the gastrointestinal tract.

**Figure 2:**
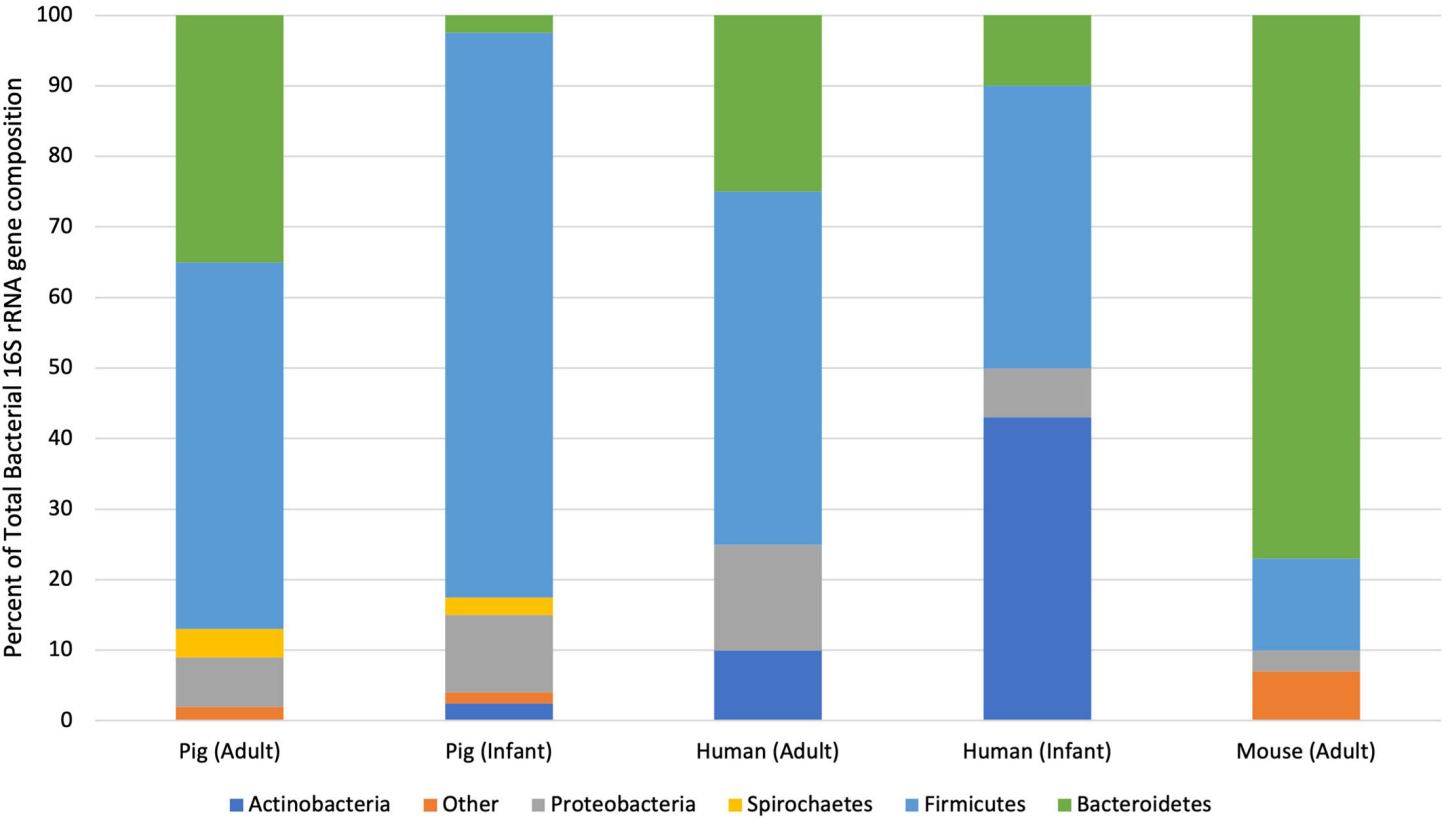
Taxonomic distribution of bacterial phyla from pigs, humans and mice at different life stages. This graph illustrates the percent of sequences assigned to each bacterial phylum isolated through 16S rRNA sequencing of pooled fecal samples isolated from healthy individuals of each species (33, 36, 37).

Given this sampling limitation, most comparative studies have been limited to the large intestines, namely the colon (Figure 2). Under natural conditions, more than 90% of the bacteria in the colon of adult humans and pigs are within one of two phyla: Firmicutes or Bacteroidetes (27, 30, 31, 33, 34, 38). Although there is slight variation in bacterial genus and species due to species specificity, shared bacterial physiology and metabolism within these phyla solidify the adult pig as a feasible model of the human colonic microbiota in health.

Species divergence becomes more readily apparent when considering the large intestinal microbiota of neonatal pigs and human infants. Large intestinal content and feces isolated from adults and infants contain significantly more Actinobacteria than adult or neonatal pigs (33, 39). In fact, the large intestines of formula- and breast-fed infants are dominated by Actinobacteria. This discrepancy between wildtype neonatal pigs and human infants can be mitigated through the use of increasingly-available, gnotobiotic animals, which will be further discussed in subsequent sections. Wildtype piglets may remain relevant models of the infant gut microbiota, however, given their shared alternations in the intestinal microbiota following the introduction of solid food (40). Therefore, wildtype neonatal pigs can be used to examine the pathogenesis and potential therapies for dietary and environmental perturbations on the intestinal microbiota of infants as long as relative alterations in specific bacterial genera and species are interpreted with care.

Although the large intestines of adults and mature pigs are dominated by the same two phyla, interspecies variability is further elucidated by comparing the bacterial genera isolated from those phyla. Within Bacteroidetes, the most abundant genus in the human intestines is *Bacteroides* while that in pig intestines is *Prevotella* (26). At 10 weeks-of-age, *Prevotella* represents up to 30% of the microbiota of the pig colon. As the pig reaches 22-weeks-old, however, relative numbers of *Prevotella* species drop to 4% and relative numbers of Anaerobacter sp., which are in the Firmicutes phylum, increase (33, 41). This steady decline in Bacteroidetes and increase in Firmicutes is mirrored in human infants over their first 4 months of life (41, 42). Therefore, although there are select differences in bacterial genera and species between pig and human intestines, shared bacterial phyla likely trigger comparable physiologic developments and establish similar symbiotic relationships.

One of the most conspicuous dissimilarities between the human and pig microbiota is the presence of specific microorganisms that are unique to pigs. In fact, both sow-reared and formula-fed piglets have greater intestinal microbial diversity than human infants (39). Low numbers of *Fusobacterium* are detected in the feces of neonatal pigs but not breast-fed infants (39). Significantly more *Lactobacilli, Spirochetes* and *Streptococci* are isolated from the porcine intestines than the human intestines at any age (26, 33, 41). A high percentage of the porcine ileal microbiota is represented by Proteobacterium, which is not reported in the human ileum (30). However, large numbers of Proteobacteria have be isolated from the feces of breast-fed infants (39). This being said, aforementioned limitations on sample collection presumably limit researchers' ability to fully characterize every bacterium within the human intestines. Identification of certain gastrointestinal commensals in pigs but not humans may be attributable to sampling techniques that are feasible in the former but not the latter.

The stark differences among the environments of humans, pigs and rodents must also be considered as a potential driver of these variations. Coprophagy is considered to be normal behavior of pigs and rodents. In piglets, the influence of sanitary conditions and coprophagia on the gastrointestinal microbiota has been well established (43, 44). Coprophagia of the sow's feces may in fact benefit piglets as a source for commensal microbes that foster the development of the piglet's microbiome. Considering the commonality of coprophagia in pigs, many researchers have turned to germ free, gnotobiotic or even humanized pigs, all of which are further discussed below.

Despite select dissimilarities in the intestinal microbiota of pigs and humans, the alluring nature of the pig as a model for the human intestinal microbiota is largely attributable to the fact that the pig is the best model we currently have. While the laboratory animal population across the world remains dominated by rodents, anatomic and physiologic dissimilarities between the human and rodent gastrointestinal tracts promote significant interspecies variation in the gut microbiota (23, 45). Nearly 85% of the bacteria genera isolated from the mouse gut is not present within the human, which immensely overshadows aforementioned variations in intestinal microbiota of pigs and humans (46).

## Cutting-Edge Techniques are Available in Pig Models

The pig's potential for high impact, translational research is further exemplified by the vast number of laboratory techniques that have been adapted to the pig. One of the biggest advantages of pig is its relative size compared to other traditional laboratory animals such as mice and rats. The pig's large gastrointestinal tract equates to increased surgical access and manipulation as well as experimental tissue volume. Furthermore, standardization of pig care and surgery permits even a novice researcher to easily expand their studies to include pig models (47–50).

Paralleling this expanse in laboratory techniques, there has been a sharp rise in the type of available pig models. The pig genome has been fully sequenced, leading to the emergence of genetically-modified pigs. Genetically-modified pigs are stronger translational models than rodents given that the human genome is more closely related to the pig than to the mouse or rat (51–53). Such models can strengthen our understanding of disease pathogenesis by introducing the ability to knock-down or knock-out genes as well as artificially tag specific cell populations and proteins so that they can be traced along the course of disease. Therefore, observations elucidated from these pigs can better inform putative disease therapies in human medicine.

In addition to genetically-modified pigs, many researchers are introducing germ-free, gnotobiotic and human-microbial associated (HMA) pigs to their experimental design. Through elimination of the intestinal microbiota in germ-free pigs, investigators may track variances in disease pathogenesis and consequently infer disease association or correlation with specific gut commensals. This being said, germ-free pigs have shorter small intestines, shorter crypts, longer villi and smaller Peyer's patches compared to conventionally-raised pigs (39). These anatomic variances, along with associated alterations in intestinal physiology and immunology, force researchers to question the translatability of germ-free pigs. Gnotobiotic pigs are arguably more powerful models of gastrointestinal disease because they permit direct manipulation of the intestinal microbiota such that investigators can characterize disease progression in the presence of select microbes. One emerging subset of gnotobiotic pigs, HMA pigs, are exceptionally noteworthy and will be discussed further within the following section.

## Important Differences Between the Human and Porcine Intestinal Microbiota May Be Surpassed Through Human-Microbial Associated Pigs

Several laboratories have successfully “humanized” the intestinal microbiota of animal models by inoculating germ-free animals with microorganisms isolated from the human intestines (11). Human microbiota-associated piglets have been established using inocula from infants, children and adults (40, 54, 55). The gut microbiota from these recipient HMA piglets is more similar to that of the human donor than that of conventionally-raised piglets. Furthermore, age-related microbial succession in HMA piglets mirrors that observed within the human donors (40).

Notably, bacteria from the Actinobacteria phyla, namely *Bifidobacteria*, successfully colonize HMA piglets and reach population densities similar to those in humans (40). Therefore, although the intestinal microbiota of conventionally-raised piglets diverges from that of infants due to the absence of naturally occurring Acintobacteria, the intestinal microbiota of HMA-piglets can be manipulated such that it closely emulates that of humans. Attempts to create HMA models in other animal species have not been as fruitful. The predominant bacterial genera of the human intestines, including Lactobacillus and Bifidobacterium, do not successfully colonize the gut of mice and zebrafish following inoculation (56–58). Therefore, not all HMA animals are powerful models of the human intestinal microbiota and something within the pig, perhaps the similarities in gastrointestinal anatomy and physiology, supports successful colonization of human bacteria.

The strength in the HMA pig model stems from the vast number of questions that can be gleaned through its integration in experimental design. As probiotics and prebiotics continue to gain momentum while synbiotics and postbiotics gain traction in the world of dietary supplementation, HMA pig models may be key to evaluating the impact of these compounds on the microbiota (59–61). Long-term studies of HMA pigs can therefore overcome the limitations in sample collection from the intestinal lumen of humans, thereby allowing further study of microbial community succession and biogeography in infants to adults. Additionally, it will be interesting to use HMA pigs to investigate whether key microbial activities can be transferred to recipient animals. This may inform future treatment modalities for gastrointestinal disorders that are associated with microbiota derangements.

## With Impactful Models Comes Impactful Costs

One of the biggest limitations of pig models is their relative cost of model acquisition and maintenance. In the Unites States, the *per diem* housing cost for rodents typically ranges between $1-3 USD while for a pig is up to $19 USD depending on the facility^1^. Given that the lifespan of most laboratory rodents is around 2–3 years and that of pigs is around 20 years, accruing costs of pig colony management may limit the feasibility of long-term, prospective studies. Even a single finishing pig, which is between 4-months and 1-year-old, is up to 30 times the cost of an adult laboratory mouse or rat (personal communication with Dr. Jack Odle, North Carolina State University). Despite the previously discussed power of gnotobiotic and HMA pig models, the limited number of laboratory facilities that can house large animals significantly potentiates costs. One litter of gnotobiotic pigs is estimated to cost around $25,000 and the cost of a 9-month study on 2 gnotobiotic litters is around $350,000 (personal communication with Dr. Michael Oglesbee, The Ohio State University). While gnotobiotic mice are certainly not cheap at $500 each, they are significantly less expensive than their porcine counterpart^2^.

## Concluding Remarks and Future Directions

As gastrointestinal researchers continue to embrace the pig model and HMA pigs become increasingly accessible, emerging translational studies will divulge the pathogenesis of and putative therapies for diseases characterized by or associated with intestinal dysbiosis. Pig models have already proven advantageous in the study of microbiota-associated diseases such as necrotizing enterocolitis of infants, which is a complication of preterm, very-low birth weight infants that has been associated with bacterial colonization of the intestines (62–70). Particularly now that the porcine intestinal microbiota has been fully characterized through multiple life stages, gastrointestinal researchers can easily track changes in the microbial composition and make direct associations between these changes and disease (28, 38, 41, 71). Compounding these studies with investigations into potential preventative and therapeutic interventions will undoubtedly uncover exciting, translational advances that will benefit humans and animals alike.

## Author Contributions

ER: manuscript generation. AB and AZ: editing and revising. All authors contributed to the article and approved the submitted version.

## Funding

ER was supported by a COHA Translational Fellowship funded by U01 TR002953.

## Conflict of Interest

The authors declare that the research was conducted in the absence of any commercial or financial relationships that could be construed as a potential conflict of interest.

## Publisher's Note

All claims expressed in this article are solely those of the authors and do not necessarily represent those of their affiliated organizations, or those of the publisher, the editors and the reviewers. Any product that may be evaluated in this article, or claim that may be made by its manufacturer, is not guaranteed or endorsed by the publisher.

## Footnotes

^1^NCSU CVM Laboratory Animal Resources. https://cvm.ncsu.edu/lar-cost/

^2^Taconic Biosciences Inc. https://www.taconic.com/mouse-model/black-6-b6ntac
